# Analysis of microbial diversity and community structure of rhizosphere soil of *Cistanche salsa* from different host plants

**DOI:** 10.3389/fmicb.2022.971228

**Published:** 2022-08-15

**Authors:** Ailing Liu, Yuxia Li, Qiqi Wang, Xinrui Zhang, Jie Xiong, Yang Li, Yonghui Lei, Yanfei Sun

**Affiliations:** ^1^College of Life Sciences, Shihezi University, Shihezi, China; ^2^Department of Plant Protection, College of Agriculture, Shihezi University, Shihezi, China

**Keywords:** *Cistanche salsa*, diversity, high-throughput sequencing, soil properties, rhizosphere soil

## Abstract

Host plants influence rhizosphere microorganism composition through root secretions, and rhizosphere associated microorganisms influence Cistanche seeds germination. At present, little is known about effects of different host plants on soil bacteria and fungi in the rhizosphere of *Cistanche salsa*. High-throughput sequencing was used here to reveal the similarities and differences in the structural composition of the soil microbial community of *C. salsa* from six host plants (i.e., *Halocnemum strobilaceum*, *Atriplex patens*, *Kalidium foliatum*, *Caroxylon passerinum*, *Anabasis aphylla*, *Krascheninnikovia ceratoides*). We discovered that *Krascheninnikovia ceratoides*-parasitizing *C. salsa* (YRCR6) had the highest diversity of rhizosphere bacterial communities, and *Anabasis aphylla -*parasitizing *C. salsa* (YRCR5) had the highest diversity of rhizosphere fungal communities. Fungal communities were more influenced by the host plant than bacterial communities. In addition, we discovered certain rhizosphere microorganisms that may be associated with Cistanche seeds germination, including *Mortierella, Aspergillus alliaceus*, and *Cladosporium*, which are account for a relatively high proportion in *Halocnemum strobilaceum*, *Atriplex patens* and *Anabasis aphylla -*parasitizing *C. salsa*. Redundancy analysis results also revealed that AP, HCO_3_^–^, pH, Ca^2+^, SO_4_^2–^, and K^+^ had a highly significant impact on the bacterial community structure (*P* < 0.01), while pH and SO_4_^2–^ had a significant impact on the fungal community structure (*P* < 0.05). Conclusively, differences were noted in the structure of rhizosphere bacterial and fungal communities of *C. salsa* parasitizing different plants in the same habit and the difference may be related to the host plant. This result can provide a new ideas for the selection of host plants and the cultivation of *C. salsa*.

## Introduction

Soil microorganisms are directly involved in processes such as plant nutrient extraction and soil nutrient cycling and are vital drivers of plant variety and production in terrestrial ecosystems. Rhizosphere microorganisms use plant rhizosphere secretions as a source of nutrients and interact with plants to influence their growth (Bulgarelli et al., 2012). In addition, the release of root exudates is affected by plant cultivar. Different plants possess different root microbial communities, which may lead to changes in microbial functions in the rhizosphere soil (Bais et al., 2006). According to previous study, rhizosphere microorganisms play a significant role in boosting the production of plant root secretions, nutrient intake by the host, and increasing the parasitic plant’s parasitism rate (Akiyama and Hayashi, 2006). Therefore, adequate study of rhizosphere soil microorganisms can help in improving plant nutrition and enhancing nutrient utilization (Zhu et al., 2017).

Rhizosphere microorganisms affect the growth of plants (Gonsior et al., 2004), especially on perennial holoparasite plants like *Cistanche salsa* (Lugtenberg and Kamilova, 2009; Marques et al., 2014). *C. salsa* is a medicinal plant belonging to the family Orobanchaceae, and its host plants are mainly sand protection plants such as *Kalidium foliatum*, *Reaumuria soongorica*, *Nitraria tangutorum*, *Salsola passerine Bunge*, *Achnatherum splendens* (Wen, 2008). All of *C. salsa*’s nutrients come from the host plant, and once a parasitic relationship is established between *Cistanche* and its host plant, they can be considered the same biological system (Li et al., 2013). Therefore, our study can be considered as a study of the rhizosphere soil microbiome of this parasitic system. *C. salsa* has a high medicinal value and is known as the “desert ginseng” (Wu et al., 2019). At present, numerous bioactive substances with crucial medicinal and edible values have been extracted from *C. salsa*. These substances can improve memory, act as a laxative, and enhance kidney function (Trampetti et al., 2019). For these reasons, the market demand for *C. salsa* has increased dramatically, leading to over-collection and the endangerment of wild *C. salsa* (Wang et al., 2017). In addition, the low parasitism rate and seed germination rate of *C. salsa* limit its growth and development, and therefore, artificial cultivation becomes difficult. Previous studies have shown that soil microorganisms affect the seed germination of parasitic plants through their metabolism. For example, *Fusarium* spp., *Rhizobium* spp., *Aspergillus alliaceus*, and *Pseudomonas* spp. reduce the parasitism of *Cistanche* seeds by affecting their germination (Jie et al., 2018). Therefore, soil microorganisms are particularly important during the germination process of *C. salsa*. Host plants directly affect the growth and development and genetic characteristics of *Cistanche*, and optimal host selection in the artificial cultivation of *Cistanche* is a crucial method for improving the quality and yield of *Cistanche* (Wang et al., 2009). Fungi in symbiosis with the host plant influence the seed germination of parasite plants. Different plants possess different symbiosis fungi (Van der Heijden and Kuyper, 2001). For example, *Rhizophagus irregularis* and *Gigaspora rosea* reduced the seed germination rate of *Orobanche cumana* (Louarn et al., 2012). Similarly, the host plant affects the germination of *Cistanche* to some extent. Thus, both rhizosphere soil microorganisms and host plants play a major role in the growth and development of *C. salsa*.

Partial information about the rhizosphere soil microorganisms of *C. salsa* is available in the literature. Sun et al. (2020a) used 16S rDNA amplicon sequencing to reveal the characteristics of soil bacterial communities of different ecotypes *C. salsa* and found that the soil microbial communities of different ecotypes *C. salsa* were significantly different, and identified the core microorganisms that can distinguish three different ecotypes *C. salsa*. In addition, XiuLi (2021) measured the soil microbial diversity of *C. salsa* with different parasitism based on 16S rDNA and ITS sequencing and found that the AMF were crucial factors affecting the germination and parasitism of *C. salsa* seeds. However, there is no relevant literature on the characteristics of the rhizosphere microorganisms of different host plants of *C. salsa*. After a series of investigations, we found that *C. salsa* was widely distributed in Qapqal County, Xinjiang, with a high diversity of host plants. This provides us with an opportunity to study the rhizosphere microbial distribution of *C. salsa* with different host plants. We found six *C. salsa* parasitized on different host plants in the same saline areas of Qapqal County (*Halocnemum strobilaceum, Atriplex patens, Kalidium foliatum, Caroxylon passerinum, Anabasis aphylla, Krascheninnikovia ceratoides*). We performed high-throughput sequencing on rhizosphere soil as well as physical and chemical indicator measurements on the soil surrounding the roots of the *C. salsa* parasitic system. To the best of our knowledge, this is the first study to investigation the diversity of bacteria and fungi in the rhizosphere soil of *C. salsa* with different host plants in the same environment in Xinjiang. The results of this study provide insight into the variations in the rhizosphere soil microbial community of *C. salsa* parasitizing on different host plants, speculates on the potential relationship between *C. salsa* parasitic system, soil microorganisms and soil physicochemical properties, and provides new ideas for the selection of host plants in the artificial cultivation of *C. salsa*.

## Materials and methods

### Sampling and processing

In June 2018, six *C. salsa* in flowering period with different host plant were found in the same saline-alkali land of Qapqal county(43°50′26″ N and 81°9′04″E). The six host plants were *Halocnemum strobilaceum* (YRCR1), *Atriplex patens* (YRCR2), *Kalidium foliatum* (YRCR3), *Salsola passerine Bunge* (YRCR4), *Anabasis aphylla* (YRCR5), and *Ceratoides latens* (YRCR6) (Supplementary Figure 1). The local annual average temperature and precipitation were 9.1°C and 245 mm, annual rainfall evaporation is 163 mm, climate type are arid temperate continental climate, soil texture are salinized sierozem and belong to light saline-alkali soil. Rhizosphere soil was collected from six *C. salsa* parasitic system using the five-point method. A sterilized shovel was used to dig down from about 10 cm away from the *C. salsa* until find the parasitic site. The host plant of *C. salsa* was then found by digging upwards with a small sterilized spade along the parasitic site. Removed the stone and plant debris attached to the parasitic roots, soil adhering to the surface of the parasitic roots was shaken off and collected in germ-free bags and immediately placed in a 4°C incubator before being transported to the laboratory stored at –80°C for high-throughput sequencing. The soil in the area of 5–10 cm around the rhizosphere of *C. salsa* parasitic system was collected (1 kg). After passing it through a sterile sieve with a pore size of 1 mm, take it back to the laboratory for air-drying process to determining the physical and chemical properties. Each sample was taken in triplicate (a total of 18 soil samples were collected).

### Physical and chemical properties of soil

This study had six groups of test soil samples, and each sample was repeated three times. A naturally dried soil sample was mixed with distilled water at a ratio of 1:2.5 (W/V), 1:5 (W/V), and 1:5 (W/V) to form three suspensions, and then the soil suspensions were shaken for 20 min. pH of the 1:2.5 (W/V) soil suspension (with the electric potential method) was measured using a pH meter (Mettle-Toledo Instruments, Shanghai, China). The electrode method was used to determine conductivity (CO) of the 1:5 (W/V) soil suspension. The soil content of the eight major ions was determined using the 1:5 (W/V) soil suspension, with HCO^3–^ in the suspension titrated by double indicator neutralization, SO_4_^2–^ by the EDTA volumetric method, Cl^–^ by silver nitrate titration, Ca^2+^ and Mg^2+^ by EDTA complex titration, and Na^+^ and K^+^ by the flame photometric method (Zhang and Gong, 2012). The potassium dichromate volumetric method–outside heating method was used to determine the organic matter (OM) content (Liu and Engineering, 2017). The Semimicro Macro Kjeldahl method and the indophenol blue spectrophotometric method were used to determine the total nitrogen (TN) and available nitrogen (AN) content of soil, respectively (Fawcett, 1954). Bray I extraction–molybdenum antimony spectrophotometric and acid soluble molybdenum antimony colorimetric assays were used to measure the total phosphorus (TP) content. The amount of available phosphorus (AP) in the soil was determined using the molybdenum blue method after extracting AP with sodium bicarbonate. The ammonium acetate extraction flame photometry analysis was performed to analyze available potassium (AK) and total potassium (TK) (Chen et al., 2016). CHCl_3_ fumigation was used to measure soil microbial biomass carbon (MBC) and microbial biomass nitrogen (MBN) (Brookes et al., 1985). Glucose produced from a sucrose substrate was colorimetrically evaluated at 508 nm using a spectrophotometer to determine invertase (INV) activity (Chen et al., 2013). Urease (UR) activity was determined by measuring the amount of ammonium released from a solution of urea (10%) and citrate buffer (pH 7) after 24 h of incubation at 37°C (Hu et al., 2014). The conversion of disodium phenyl phosphate to phenol was used to measure phosphatase (PHA) activity (Jin et al., 2016). Catalase (CAT) activity was determined by monitoring the decrease in H_2_O_2_ absorbance at 240 nm over 2 min and then applying an extinction coefficient of 40 M^–1^cm^–1^ for calculation (Abei, 1984). Nitrate reductase (NR) activity was determined by incubating soil samples at 25°C for 24 h and controls at –20°C. The released nitrates were extracted with 4 M KCl solution and colorimetrically measured at 520 nm (Schinner et al., 1996).

### DNA extraction and PCR amplification

Microbial community genomic DNA was extracted from *C. salsa* rhizosphere soil samples (0.5 g) using the EZNA^®^ Soil DNA Kit (Omega Bio-tek, Norcross, GA, United States). The NanoDrop 2000 UV-Vis spectrophotometer (Thermo Scientific, Wilmington, DE, United States) was used to determine DNA concentration and purity, and 1% agarose gel electrophoresis was used to ensure DNA quality and integrity. The bacterial 16S rRNA gene’s hypervariable region V3–V4 was amplified with primer pairs: 515F (forward primer) (5′-GTGCCAGCMGCCGCGGTAA-3′) and 806R (reverse primer) (5′-GGACTACHVGGGTWTCTAAT-3′) (Dennis et al., 2013; Xu et al., 2016). The PCR system contained 4 μL of 5 × FastPfu buffer solution, 2 μL of 2.5 mM dNTPs, 0.8 μL of 5 μM forward and reverse primers, 0.4 μL of DNA polymerase, 0.2 μL of BSA solution, and 1 μL of template DNA, and finally, double-distilled H_2_O was added to form a volume of 20 μL. The PCR was carried out on an ABI Gene Amp 9700 PCR thermocycler (ABI, California, CA, United States), with an initial denaturation at 95°C for 3 min, followed by 27 cycles of denaturation at 95°C for 30 s, annealing at 55°C for 30 s, and extension at 72°C for 45 s, and a single extension at 72°C for 10 min, before finishing at 4°C. The fungal Internal Transcribed Spacer gene was amplified with primer pairs: ITS1 (forward primer) (5′-CTTGGTCATTTAGAGGAAGTAA-3′) and ITS2 (reverse primer) (5′-GCTGCGTTCTTCATCGATGC-3′). The PCR system included 2 μL of 10 × FastPfu buffer solution, 2 μL of 2.5 mM dNTPs, 0.8 μL of 5 μM forward and reverse primers, 0.2 μL of rTaq of DNA polymerase, 0.2 μL of BSA solution, and 1 μL of template DNA, and finally, double-distilled H_2_O was added to form a volume of 20 μL. The PCR process consisted of an initial denaturation at 94°C for 5 min, followed by 30 cycles of denaturation at 94°C for 30 s, annealing at 55°C for 30 s, extension at 72°C for 1 min, and single extension at 72°C for 7 min, before finishing at 4°C. The PCR thermocycler model was the same as that used for bacterial PCR.

### Illumina MiSeq sequencing

The PCR products were separated on 2% agarose gel electrophoresis, purified using the AxyPrep DNA Gel Extraction Kit (Axygen Biosciences, Union City, CA, United States) according to the manufacturer’s instructions, and quantified using the QuantusTM Fluorometer (Promega, WI, United States); each reaction was performed in triplicate (Zhang et al., 2018). Purified amplicons were pooled in equimolar proportions and paired-end sequenced on an Illumina MiSeq PE300 platform (Illumina, San Diego, CA, United States) following the standard protocols of Meiji Biomedical Technology Co. Ltd. (Shanghai, China). The raw reads were uploaded to the NCBI Sequence Read Archive database (Accession Number: PRJNA764328).

### Processing and analyzing of sequencing data

Fastq version 0.20.0 (Chen et al., 2018) was used to demultiplex and quality filter raw sequence files, and FLASH version 1.2.7 (Magoč and Salzberg, 2011) was used to merge them. The 300-bp reads were truncated at any site receiving an average quality score of < 20 over a 50-bp sliding window. The truncated reads shorter than 50 bp and the reads containing ambiguous characters were removed. Overlapping sequences greater than 10 bp were assembled according to their overlapped sequence, and those that could not be assembled were eliminated. The overlap region’s maximum mismatch ratio was 0.2. The samples were distinguished on the basis of the barcode and primers, the sequence orientation was modified, the number of mismatches allowed by the barcode was set to 0, and the maximum number of primer mismatches was set to 2. With a 97% similarity cutoff, UPARSE version 7.1 was used to cluster operational taxonomic units (OTUs), and chimeric sequences were discovered and discarded (Stackebrandt and Goebel, 1994; Edgar, 2013). OTU-based taxonomy information can be used to conduct statistical analyses of the community structure at each classification level. RDP Classifier version 2.2 was used to assess the taxonomy of each OTU representative sequence against the 16S rRNA database (Silva 138) and the ITS database (Unite 8.0) with a confidence threshold of 0.7 (Wang et al., 2007; Jing et al., 2018).

### Statistical analysis

Mothur v.1.30.2 was used to calculate alpha diversity, including the observed richness (Sobs), Chao1 estimator, ACE index, Shannon diversity index, and Simpson index (Schloss et al., 2009). The rarefaction curve based on the alpha diversity index was constructed using Past 4.0. Venn diagrams were generated using the Venn Diagram program (Chen and Boutros, 2011). The community ecology package was used to perform the principal coordinate analysis (PCoA), the Vegan 2.0 package was used to generate a PCoA figure. Redundancy analysis (RDA) were plotted using Canoco 5.0. Heatmap figures were generated in Vegan 2.0 in R programming language (Hong et al., 2015). Linear discriminant analysis (LDA) effect size (LEfSe) using Galaxyonline analytics platform^1^ to perform. SPSS Statistics v25.0 was used for analyzing data regarding soil physical and chemical parameters (IBM, NY, United States). All values are presented as mean ± standard error (mean ± SE).

## Results

### Soil physicochemical properties

To explore how soil environmental factors affect soil bacterial communities, we measured soil pH, OM, TN, TP, TK, AN, AP, and AK (Table 1). At the same time, we tested CO and major ions of the soil, including Cl^–^, SO_4_^2–^, Ca^2+^, K^+^, Mg^2+^, Na^+^, HCO_3_^–^ (Table 2). Soil MBC and MBN content and CAT, UR, PHA, INV, NR activities were also measured (Table 3). The rhizosphere soil pH of different host plants fluctuated between 8.05 and 8.31, and significant differences in pH were noted between the samples (*P* < 0.05). Except for YRCR4 and YRCR6, the content of OM and TN had significant differences between samples, and YRCR4 and YRCR6 were significantly greater than other samples (*P* < 0.05). TP content was not significantly different between groups except for YRCR4 and YRCR6, and YRCR4 and YRCR6 TP content was significantly higher than the other samples. TK and AK content in YRCR6 was markedly higher than that in the other five samples. AN content was considerably higher in YRCR4 than the other five samples. AP content was significantly higher in the YRCR5 rhizosphere soil than other samples. The final results of the analysis showed that *C. salsa* parasitizing *Krascheninnikovia ceratoides* (YRCR6) and *Caroxylon passerinum* (YRCR4) had higher OM, TN, TP, TK, AN, and AK content in the rhizosphere soil. The soil rhizosphere of *C. salsa* parasitizing *Anabasis aphylla* (YRCR5) had the greatest AP content, this is anomalous among the organic indicators measured. By analyzing the relationship between CO and salinity of the saturated leachate of soil, we collected six plants with rhizosphere soil salinity in the order of YRCR1, YRCR4, YRCR3, YRCR6, YRCR2, and YRCR5 from the highest to the lowest. Based on the content of each ion in the soil, the rhizosphere soil was found to be mainly dominated by sulfate, sodium, chloride, and calcium salts. The analysis of measured ions showed the same trend for Na^+^ and Cl^–^ content in each sample and for CO, with significant differences observed among all samples (*P* < 0.05). The contents of other ions exhibited variable trends among the samples, but were the lowest in YRCR5. The enzyme activity assay showed high CAT, UR, PHA, and INV activities in YRCR6.

**TABLE 1:** Physical and chemical characteristics of different soil samples.

Sample ID	PH	OM(g/kg)	TN(g/kg)	TP(g/kg)	TK(g/kg)	AN (mg/kg)	AP (mg/kg)	AK (mg/kg)
YRCR1	8.22 ± 0.00 c	11.93 ± 0.54 c	0.70 ± 0.00 d	0.94 ± 0.02 c	17.68 ± 0.07 d	29.74 ± 0.32 d	2.14 ± 0.23 d	545.10 ± 3.58 e
YRCR2	8.14 ± 0.01 d	9.65 ± 0.49 d	0.63 ± 0.01 e	0.96 ± 0.01 c	19.41 ± 0.39 b	29.60 ± 0.18 d	2.32 ± 0.08 cd	576.13 ± 3.11 d
YRCR3	8.31 ± 0.01 a	14.71 ± 0.17 b	0.82 ± 0.01 b	0.93 ± 0.01 c	18.27 ± 0.08 c	34.66 ± 0.35 c	2.31 ± 0.06 cd	820.00 ± 5.20 b
YRCR4	8.05 ± 0.01 f	16.18 ± 0.09 a	0.95 ± 0.00 a	1.04 ± 0.00 b	19.73 ± 0.17 b	44.02 ± 0.73 a	2.53 ± 0.14 c	796.33 ± 4.16 c
YRCR5	8.08 ± 0.01 e	11.55 ± 0.21 c	0.75 ± 0.01 c	0.94 ± 0.01 c	16.91 ± 0.25 e	34.78 ± 0.71 c	4.29 ± 0.14 a	534.03 ± 6.79 f
YRCR6	8.30 ± 0.01 b	15.47 ± 0.77 ab	0.95 ± 0.02 a	1.07 ± 0.02 a	21.23 ± 0.27 a	38.00 ± 0.93 b	3.01 ± 0.07 b	879.67 ± 8.39 a

OM, organic matter; TN, total nitrogen; TP, total phosphorus; TK, total potassium; AN, available nitrogen; AP, available phosphorus; AK, available potassium. YRCR1, C. salsa parasitic on Halocnemum strobilaceum; YRCR2, C. salsa parasitic on Atriplex patens.; YRCR3, C. salsa parasitic on Kalidium foliatum; YRCR4, C. salsa parasitic on Caroxylon passerinum.; YRCR5, C. salsa parasitic on Anabasis aphylla.; YRCR6, C. salsa parasitic on Krascheninnikovia ceratoides.

Data was shown by the average of three replicates and their standard deviation. Different letters following after the data indicated significant differences (P < 0.05) based on the Kruskal–Wallis test.

**TABLE 2:** Electrical conductivity and major ions of soil samples.

Sample ID	EC (us/cm)	Cl^–^ (mg/g)	SO_4_^2–^(mg/g)	Ca^2+^ (mg/g)	K^+^ (mg/g)	Mg^2+^ (mg/g)	Na^+^ (mg/g)	HCO_3_^–^(mg/g)
YCRC1	2333.33 ± 15.28 a	6.09 ± 0.17 a	8.44 ± 0.09 b	3.35 ± 0.08 a	0.25 ± 0.01 d	0.45 ± 0.08 a	4.36 ± 0.11 a	0.13 ± 0.00 d
YCRC2	1441.33 ± 5.13 e	1.58 ± 0.08 e	7.72 ± 0.35 c	2.77 ± 0.08 cd	0.28 ± 0.02 c	0.12 ± 0.01 df	1.65 ± 0.09 e	0.17 ± 0.00 a
YCRC3	1873.67 ± 34.53 c	3.35 ± 0.04 c	9.38 ± 0.53 a	2.83 ± 0.01 bc	0.38 ± 0.00 a	0.18 ± 0.00 cd	3.27 ± 0.02 c	0.11 ± 0.00 e
YCRC4	2080.00 ± 10.00 b	4.80 ± 0.02 b	8.17 ± 0.01 bc	3.43 ± 0.02 a	0.34 ± 0.01 b	0.35 ± 0.01 b	3.46 ± 0.02 b	0.14 ± 0.00 c
YCRC5	1075.00 ± 5.29 f	0.09 ± 0.01 f	7.61 ± 0.44 c	2.73 ± 0.03 d	0.23 ± 0.00 d	0.19 ± 0.00 c	0.35 ± 0.01 f	0.11 ± 0.00 e
YCRC6	1738.33 ± 4.04 d	2.10 ± 0.02 d	7.64 ± 0.06 c	2.89 ± 0.03 b	0.35 ± 0.02 b	0.09 ± 0.01 f	2.65 ± 0.01 d	0.16 ± 0.00 b

EC, electrical conductivity.

The major ions measured include: Cl^–^; SO_4_^2–^; Ca^2+^; K^+^; Mg^2+^; Na^+^; HCO_3_^–^. YRCR1, C. salsa parasitic on Halocnemum strobilaceum; YRCR2, C. salsa parasitic on Atriplex patens.; YRCR3, C. salsa parasitic on Kalidium foliatum; YRCR4, C. salsa parasitic on Caroxylon passerinum; YRCR5, C. salsa parasitic on Anabasis aphylla.; YRCR6, C. salsa parasitic on Krascheninnikovia ceratoides. Data was shown by the average of three replicates and their standard deviation. Different letters following after the data indicated significant differences (P < 0.05) based on the Kruskal–Wallis test.

**TABLE 3:** Soil MBC and MBN content and CAT, UR, PHA, INV, and NR activities.

Sample ID	CAT(mg/g)	UR(mg/g)	PHA(mg/g)	INV(mg/g)	NR(ug/g)	MBC (mg/g)	MBN (mg/g)
YRCR1	2.933 ± 0.0026 d	0.297 ± 0.0044 e	0.364 ± 0.0044 f	5.922 ± 0.0078 b	0.101 ± 0.0540 ab	0.050 ± 0.0106 d	8.963 ± 0.0046 e
YRCR2	3.201 ± 0.0030 c	0.356 ± 0.0076 d	0.423 ± 0.0040 d	3.429 ± 0.0051 f	0.081 ± 0.0036 a	0.130 ± 0.0262 c	10.800 ± 0.1212 c
YRCR3	3.201 ± 0.0053 c	0.500 ± 0.1370 b	0.376 ± 0.0026 e	5.386 ± 0.0112 c	0.028 ± 0.0036 c	0.170 ± 0.0165 b	18.000 ± 0.5580 a
YRCR4	3.289 ± 0.0085 b	0.291 ± 0.0026 e	0.536 ± 0.0061 c	4.867 ± 0.0053 d	0.044 ± 0.0030 abc	0.160 ± 0.0370 bc	14.222 ± 0.0306 b
YRCR5	2.934 ± 0.0017 d	0.453 ± 0.0053 c	0.687 ± 0.0030 b	4.252 ± 0.0044 e	0.037 ± 0.0026 bc	0.130 ± 0.0017 c	10.267 ± 0.2393 d
YRCR6	3.817 ± 0.0157 a	0.609 ± 0.0078 a	0.86 ± 0.0044 a	9.925 ± 0.0098 a	0.085 ± 0.0052 a	0.220 ± 0.0010 a	14.444 ± 0.0624 b

CAT, catalase activity; UR, urease activity; PHA, phosphatase activity; INV, invertase activity; NR, nitrate reductase activity; MBC, microbial biomass carbon; MBN, soil microbial biomass nitrogen; YRCR1, C. salsa parasitic on Halocnemum strobilaceum; YRCR2, C. salsa parasitic on Atriplex patens; YRCR3, C. salsa parasitic on Kalidium foliatum; YRCR4, C. salsa parasitic on Caroxylon passerinum; YRCR5, C. salsa parasitic on Anabasis aphylla; YRCR6, C. salsa parasitic on Krascheninnikovia ceratoides.

Data was shown by the average of three replicates and their standard deviation. Different letters following after the data indicated significant differences (P < 0.05) based on the Kruskal–Wallis test.

### Alpha diversity analysis of sequencing data

After read-quality filtering, Illumina-based analysis of the hypervariable V3-V4 region of the bacterial 16S rRNA gene yielded 1207863 high-quality reads, and the analysis of the ITS1 region of the fungi produced 1230526 high-quality reads. Each sample included an average of 67,104 and 68,363 bacterial and fungal reads, respectively. The average read length for bacteria was 253 bases and for fungi was 245 bases. The rhizosphere bacterial community contained 45 phyla, 121 classes, 292 orders, 491 families, 854 genera, 1451 species, while the rhizosphere fungal community contained 9 phyla, 29 classes, 60 orders, 139 families, 247 genera, 368 species based on the minimum sample sequence. A total of 4566 OTUs for bacterial diversity and 819 OTUs for fungal diversity were generated for each sample based on 97% similarity. The Shannon rarefaction curves of bacteria and fungi tended to be smooth, reflecting the availability of sufficient sequencing data (Supplementary Figure 2). The coverage valuation of both bacteria and fungi reached 99%, indicating that only a very few sequences were not detected in the samples (Table 4). Therefore, the alpha diversity index of bacteria and fungi in the sequenced samples represent the abundance and diversity of bacteria and fungi. Our analysis showed that the highest bacterial diversity was found in YRCR6 and the lowest bacterial diversity was found in YRCR5. No remarkable differences (*P* > 0.05) were noted in bacterial diversity among the four samples YRCR1, YRCR2, YRCR3, and YRCR4. Shannon index shown that YRCR5 have highest fungal community diversity, which was significantly highest from that of *C. salsa* YRCR4 andYRCR6 (*P* < 0.05). In addition, no significant differences in fungal community diversity were noted among the other four samples (*P* > 0.05). The abundance of the bacterial community of YRCR3 was significantly lower than that of other samples, according to the Chao 1 index and ACE index analyses, and no statistically significant difference was observed in bacterial community abundance across the remaining five samples. YRCR6 had the highest fungal community abundance, followed by YRCR2. Although no significant difference in abundances was noted between YRCR6 and YRCR2 (*P* > 0.05), the abundances in these samples were significantly higher than those in the other samples. In the remaining four samples, no significant differences were noted in the abundance of fungal communities (*P* > 0.05). Overall, there were difference in rhizosphere soil bacterial and fungal communities of *C. salsa* parasitizing different plants. The bacterial community diversity and abundance was maximum in the rhizosphere soils of *C. salsa* parasitizing *Krascheninnikovia ceratoides* (YRCR6). The rhizosphere soil of *C. salsa* parasitizing *Anabasis aphylla* (YRCR5) had the most diversity of fungal communities, whereas that of *C. salsa* parasitizing *Krascheninnikovia ceratoides* (YRCR6) had the highest abundance.

**TABLE 4:** Alpha diversity indices of bacteria and fungi in rhizosphere soil of *C. salsa* with different host plant.

Sample ID	Sobs		Chao1		Ace		Shannon		Simpson		Coverage(%)	
							
	Bacteria	Fungi	Bacteria	Fungi	Bacteria	Fungi	Bacteria	Fungi	Bacteria	Fungi	Bacteria	Fungi
YRCR1	2385.00 ± 81.07 abc	155.67 ± 9.81 b	2944.14 ± 50.76 a	164.62 ± 8.73 b	2929.52 ± 76.93 a	163.32 ± 8.86 b	5.90 ± 0.15 b	3.38 ± 0.59 a	0.01 ± 0.00 b	0.10 ± 0.08 ab	99.10	99.99
YRCR2	2434.67 ± 34.27 ab	201.67 ± 29.84 a	2940.99 ± 86.16 a	207.79 ± 31.86 a	2938.21 ± 70.65 a	205.88 ± 29.75 a	5.89 ± 0.04 b	3.41 ± 0.50 a	0.02 ± 0.01 b	0.10 ± 0.07 ab	99.12	99.98
YRCR3	2022.00 ± 70.93 d	152.00 ± 7.81 b	2530.35 ± 44.68 b	158.44 ± 13.62 b	2539.09 ± 47.38 b	159.39 ± 14.36 b	5.68 ± 0.11 c	2.90 ± 0.56 ab	0.02 ± 0.01 b	0.18 ± 0.11 ab	99.19	99.99
YRCR4	2311.00 ± 67.67 bc	149.33 ± 10.12 b	2869.09 ± 109.74 a	161.97 ± 13.52 b	2836.79 ± 69.22 a	157.57 ± 12.28 b	5.79 ± 0.11 bc	2.49 ± 0.47 b	0.02 ± 0.01 b	0.23 ± 0.08 a	99.11	99.98
YRCR5	2278.00 ± 108.02 c	169.00 ± 7.00 b	2861.53 ± 133.08 a	173.46 ± 3.83 b	2854.90 ± 118.50 a	173.11 ± 5.96 b	5.37 ± 0.14 d	3.56 ± 0.07 a	0.01 ± 0.01 a	0.07 ± 0.00 b	99.13	99.99
YRCR6	2473.00 ± 38.59 a	210.00 ± 6.24 a	2964.40 ± 26.58 a	225.72 ± 6.34 a	2926.02 ± 34.53 a	226.36 ± 6.49 a	6.22 ± 0.05 a	2.44 ± 0.05 b	0.01 ± 0.00 c	0.17 ± 0.02 ab	99.17	99.96

Sobs indices was used to evaluate the number of observable OTUs; Chao 1 and ACE indices were used to evaluate species richness; Shannon and Simpson indices were used to evaluate species diversity; Data was shown by the average of three replicates and their standard deviation.

Different letters following after the data indicated significant differences (P < 0.05) based on Kruskal–Wallis test.

YRCR1, C. salsa parasitic on Halocnemum strobilaceum; YRCR2, C. salsa parasitic on Atriplex patens; YRCR3, C. salsa parasitic on Kalidium foliatum; YRCR4, C. salsa parasitic on Caroxylon passerinum; YRCR5, C. salsa parasitic on Anabasis aphylla; YRCR6, C. salsa parasitic on Krascheninnikovia ceratoides.

### Bacterial community analysis

**FIGURE 1:**
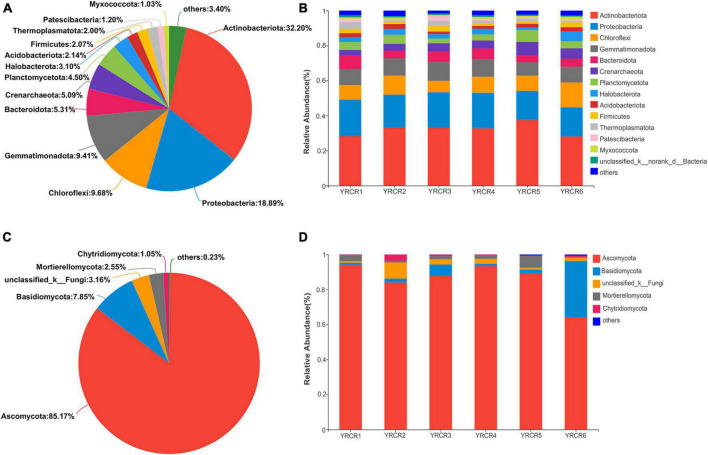
**(A)** Is the bacterial community analysis pieplot on phylum level of all samples. **(B)** Is the relative abundance of bacterial on phylum level. **(C)** Is community abundance of fungal in all samples on phylum level. **(D)** Is community abundance of fungal in each sample on phylum level.

High-throughput sequencing revealed the diversity of bacterial communities in different samples. At the phylum level, 37 phyla were identified. The phylum with relative abundance greater than 1% are *Actinobacteriota* (32.2%), *Proteobacteria* (18.89%), *Chloroflexi* (9.68%), *Gemmatimonadota* (9.41%), *Bacteroidota* (5.31%), *Crenarchaeota* (5.09%), *Planctomycetota* (4.05%), *Halobacterota* (3.10%), *Acidobacteriota* (2.14%), *Firmicutes* (2.07%), *Thermoplasmatota* (2.00%), *Myxococcota* (1.03%), and *Patescibacteria* (1.02%) (Figure 1A). However, their relative abundance varied in different host plant soils. The most abundant phylum in the six samples was *Actinobacteriota*, its abundance in the soil samples of each host plant ranged from high to low: YRCR5 (37.45%), YRCR2 (33.26%), YRCR3 (33.01%), YRCR4 (32.72%), YRCR1 (28.26%), and YRCR6 (28.12%). *Proteobacteria* was the second abundant phylum, its abundance in the soil samples of each host plant was YRCR1 (20.85%), YRCR4 (20.31%), YRCR3 (20.30%), YRCR2 (18.68%), YRCR5 (16.67%), and YRCR6 (16.65%) in the descending order (Figure 1B). Among these phyla, *Actinobacteriota, Chloroflexi, Gemmatimonadota, Bacteroidota, Halobacterota* and *Myxococcota* have significant difference in six samples (Supplementary Figure 3A).

**FIGURE 2:**
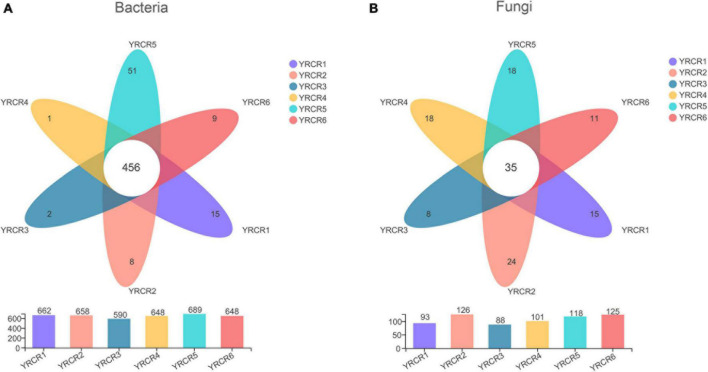
Venn diagram at the genus level of six samples. **(A)** Represents bacteria, **(B)** represents gungal. Each circle with different colors in the diagram represents a group; middle core numbers represent the number of genus common to all groups.

**FIGURE 3:**
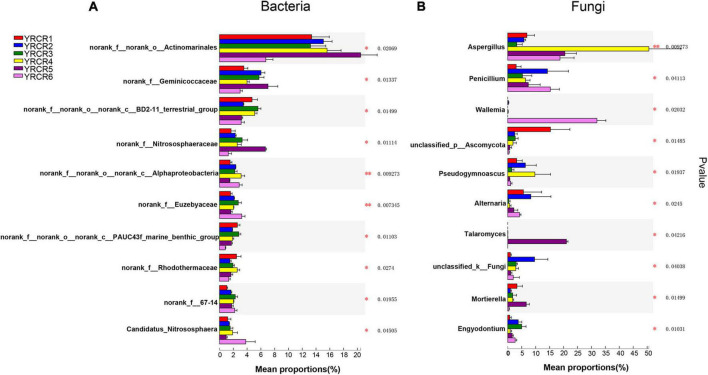
Species difference analysis of all samples on genus level. The *y*-axis represents the genus levels of species, and the *x*-axis represents the percentage of species average relative abundance in each sample group. **(A)** Is represent bacteria; **(B)** is represent Fungi. The Kruskal–Wallis rank-sum test was used to show significant differences (*: 0.01 < *P* < = 0.05, ^**^: 0.001 < *P* < = 0.01).

A total of 854 genera were identified from all rhizosphere soil samples, further analysis revealed 456 common genera in the six samples (Figure 2A). From the Venn diagram we can find that each sample has its own unique genus. However, there are 51 unique genera in YRCR5, which is much higher than several other samples. Unexpectedly, genera with relative abundance greater than 1% were found in all six samples. It indicates that the relative abundance of YRCR5 unique genera is less than 1%. The genera with relative abundance greater than 1% are shown in pieplot (Supplementary Figure 4A). The most abundant genera is *norank_f__norank_o__Actinomarinales* (14.11%), following by *norank_f__Geminicoccaceae* (4.91%), *norank_f__norank_o__norank_c__BD211_terrestrial_group* (4.19%), *norank_f__Nitrososphaeraceae* (3.01%), *norank_f__ norank_o__norank_c__Alphaproteobacteria* (2.25%), *norank _f__Euzebyaceae* (2.22%). Among them, *norank_f__ norank_o__norank_c__Alphaproteobacteria* and *norank_f __Euzebyaceae* showed extremely significant differences (*P* < 0.01) in the relative abundance among all samples. The remaining four genera differed significantly among the samples (*P* < 0.05) (Figure 3A).

### Fungal community analysis

The composition of fungal communities in all samples was simpler than that of bacterial communities. Nine fungal phyla were found in all samples, with four phyla having a relative abundance of > 1%: *Ascomycota*, *Basidiomycota*, *Mortierellomycota*, and *Chytridiomycota* (Figure 1C). Among all the phyla, *Ascomycota* was the main dominant phylum and accounted for 85.17% of all the fungal phyla. The secondary dominant phyla included *Basidiomycota*, *Mortierellomycota*, and *Chytridiomycota*, which accounted for 7.85, 2.55, and 1.05% of all fungal phyla, respectively (Figure 1D). Among them, *Ascomycota*, *Mortierellomycota* were significant different in six samples (*P* < 0.05). *Ascomycota* was higher in YRCR1 than in the other phyla, *Mortierellomycota* was higher in YRCR5 than in the other phyla (Supplementary Figure 3B).

At the genus level, we discovered 247 fungal, further analysis revealed 35 common genera in the six samples (Figure 2B). Community pieplot analysis showed the 23 genera with relative abundances of > 1% (Supplementary Figure 4B). The proportion of fungi in the top 10 genera with a relative abundance ranging from the highest to lowest were *Aspergillus* (16.94%), *Penicillium* (8.71%), *Chaetomium* (7.42%), *Sporormia* (7.05%), *Wallemia* (5.54%), *unclassified_p __Ascomycota* (3.90%), *Talaromyces* (3.70%), *Alternaria* (3.63%), *Pseudogymnoascus* (3.33%), *unclassified_k__Fungi* (3.16%). Among these genera, significant differences (*P* < 0.05) were noted across all 8 genera in all samples (Figure 3B). *Aspergillus* differed extremely significantly (*P* < 0.01) among the six samples. *Aspergillus* and *Pseudogymnoascus* were mainly present in YRCR4. *Penicillium* was mainly present in YRCR2 and YRCR6. *Alternaria* were mainly observed in YRCR2. *Mortierella* was almost exclusively present in YRCR5. *Engyodontium* was mainly found in YRCR3. It is worth noting that *Wallemia* was almost exclusively found in YRCR6, *Talaromyces* was almost exclusively found in YRCR5.

### Comparison of bacterial and fungal communities of different samples

**FIGURE 4:**
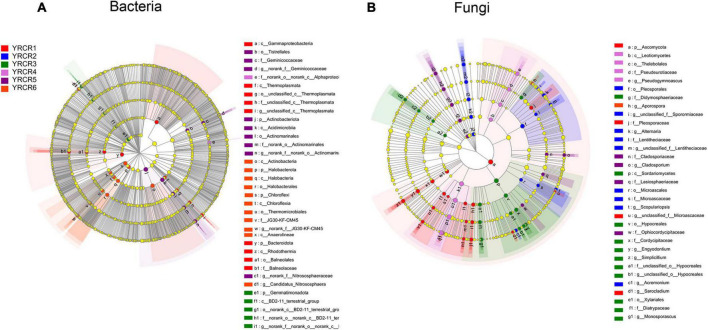
LEfSe analysis showing the different biomarkers among different ginseng cultivars rhizosphere in bacteria **(A)** and fungi **(B)**. Different colored regions represented different constituents, the diameter of each circle is proportional to the relative abundance of the taxon. The inner to outer circle corresponds to the level of the phylum to the genus.

To confirm the effect of bacterial and fungal communities on the sample, we performed LEfSe analysis for LDA scores above 4.0 (Supplementary Figure 5). The results showed a total of 36 significantly enriched bacterial taxa in the six samples (*P* < 0.05). Among them, 12 rich taxa were present in YRCR6, and it comprised most of the six samples. At the phylum level, *Chloroflexi* and *Halobacterota* were significantly enriched in YRCR6. *Actinobacteriota* was significantly enriched in YRCR5. *Gemmatimonadota* was significantly enriched in YRCR3. *Bacteroidota* was significantly enriched in YCRC1. At the genus level, *Candidatus_Nitrososphaera* was significantly enriched in YRCR6 (Figure 4A).

A total of 66 fungal taxa were significantly enriched in the six samples, and YRCR1 had 18 differentially enriched groups. At the phylum level, *Ascomycota* was the significantly enriched fungus in YRCR1. *Mortierellomycota* was the most enriched taxon in YRCR5. At the genus level, *Sarocladium* was the significantly enriched taxon in YRCR1. *Alternaria* and *Acremonium* were the significantly enriched taxa in YRCR2. *Monosporascus*, *Engyodontium*, *Simplicillium*, and *Cutaneotrichosporon* were the significantly enriched taxa in YRCR3. *Aspergillus* and *Pseudogymnoascus* were significantly enriched in YRCR4. *Mortierella* and *Cladosporium* were significantly enriched in YRCR5. *Penicillium* and *Aporospora* were significantly enriched in YRCR6 (Figure 4B).

**FIGURE 5:**
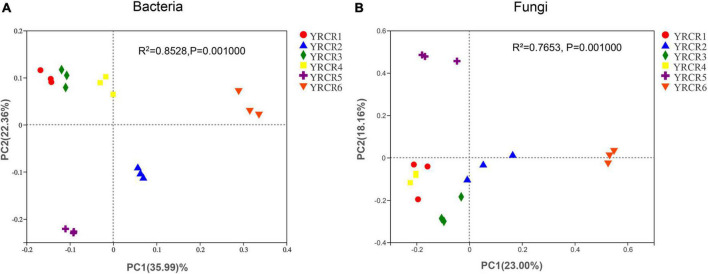
**(A)** Is principal coordinate analysis (PCoA) of the bacterial diversity. **(B)** Is principal coordinate analysis (PCoA) of the fungal diversity. The hierarchical clustering and PCoA plots were using Bary–Curtis distance method at OUT level.

Bacterial and fungal communities in all soil samples were compared separately at the OTU level by using PCoA based on the Bray–Curtis matrix algorithm and hierarchical clustering to identify parallels or variations in community composition between groups of samples. The first PCoA axis (PCoA1) explains 35.99% of the overall variation in the bacterial community, while the second PCoA axis (PCoA2) explains 22.36% of the entire variation in the bacterial community (Figure 5A). Among the six soil samples, three replicates clustered together within each sample group showed good reproducibility, and a good separation was noted between each grouped sample. Meanwhile, the principal component analysis diagram clearly showed that YRCR1, YRCR3, and YRCR4 are similar in bacterial community composition, and YRCR2 and YRCR5 are similar in bacterial community composition. Adonis analysis results showed that the *R*^2^ value (*R*^2^ = 0.83020.0.0.) was greater than 0 and tended to 1, suggesting that the differences between the sample groups were greater than those within the sample groups. A *P*-value of < 0.05 (*P* = 0.001) indicated significant differences between sample groups (Supplementary Figure 6A).

Similarly, the fungal communities were subjected to PCoA. PCoA1 and PCoA2 alone explained 23 and 18.16% of the variance, respectively (Figure 5B). Except for YRCR1, YRCR3, and YRCR4 exhibited high similarity, whereas YRCR6 exhibited a low similarity with the other five samples. Adonis analysis results showed that the *R*^2^ value was 0.7568, suggesting that the difference between the total samples was higher than that within the group. A *P*-value of < 0.05 (*P* = 0.001) indicated that the difference between the samples was significant (Supplementary Figure 6B).

### Relationship between bacterial and fungal community structures and soil properties

**FIGURE 6:**
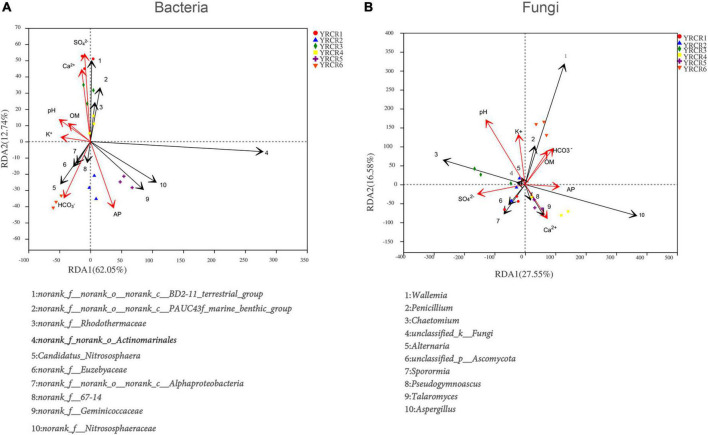
**(A)** Is RDA of the correlation between bacterial and soil physicochemical on genus level. **(B)** Is RDA of the correlation between fungal and soil physicochemical.

Redundancy analysis revealed the influence of many environmental conditions on soil microbial communities, as well as the correlation between them. RDA was performed for the top 10 bacterial genera and soil physicochemical properties. The results showed that the first RDA axis accounted for 62.05% of the total variance and the second axis accounted for 12.74% of the total variance, with a total variance of 74.79% for both axes (Figure 6A). At a 0.05 significance level, SO_4_^2–^ had a positive and significant correlation with *norank_f__norank_o__norank_c__BD-211_terrestrial_group*, *norank_f__norank_o__norank_c__PAUC43f_marine_benthic_ group*, and *norank_f__Rhodothermaceae.* Ca^2+^ exhibited a positive and significant correlation with *norank_f __Rhodothermaceae*, whereas it exhibited a considerable negative relationship with *norank_f__Geminicoccaceae* and *norank_f__Nitrososphaeraceae*. pH and K^+^ had significant positive correlations with *norank_f__Euzebyaceae* and negative correlations with *norank_f__norank_o__Actinomarinales*. In addition, like OM, K^+^ had significant positive correlations with *norank_f__67-14*, *norank_f__norank_o __norank_c__Alphaproteobacteria*, and *Candidatus_ Nitrososphaera*. HCO_3_^–^ was significantly and negatively correlated with *norank_f__Nitrososphaeraceae*, *norank_f__ norank_o__norank_c__PAUC43f_marine_benthic_group*, and *norank_f__norank_o__norank_c__BD-211_terrestrial_group*, whereas it was significantly and positively correlated with *norank_f__norank_o__norank_c__Alphaproteobacteria*. Among the environmental factors analyzed for RDA, SO_4_^2–^, AP, and HCO_3_^–^ had the greatest impact on the rhizosphere soil bacterial community structure (*P* = 0.003), followed by pH (*P* = 0.006), Ca^2+^ (*P* = 0.024), and K^+^ (*P* = 0.020). OM (*P* = 0.147) had the least effect on the bacterial community structure (Table 5).

**TABLE 5:** Significance of the soil physicochemical properties in explaining the community structure obtained from the RDA results.

Environmental factors	Bacteria		Fungi	
		
	*R* ^2^	*P*	*R* ^2^	*P*
OM	0.2406	0.147	0.223133	0.15
AP	0.599315	0.003	0.223133	0.174
pH	0.494644	0.006	0.770272	0.001
SO_4_^2–^	0.593181	0.003	0.417195	0.014
Ca^2+^	0.424395	0.024	0.212356	0.163
K^+^	0.394594	0.020	0.30736	0.078
HCO_3_^–^	0.56574	0.003	0.307182	0.085

The larger the value of R^2^ (the ratio of group variance to total variance), the more significant the difference were among the environmental factors and microbial community; P < 0.05 indicates a high reliability of the test.

Similarly, RDA was performed for the top 10 fungal genera and soil physicochemical properties, the results showed that the first RDA axis accounted for 27.55% of the total variance and the second axis accounted for 16.58% of the total variance, with a total variance of 44.13% for both axes (Figure 6B). At a 0.05 significance level, AP was markedly positively connected with *Aspergillus* and *Talaromyces* and negatively linked with *Pseudogymnoascus* and *unclassified_p__Ascomycota*. SO_4_^2–^ was significantly positively linked with *unclassified_p__Ascomycota* and significantly negatively linked with *Alternaria*, *Wallemia*, and *Penicillium*. On the contrary, HCO_3_^–^ was significantly positively correlated with *Wallemia* and *Penicillium*. pH showed a significant negative correlation with *Aspergillus*. K^+^ was significantly and positively connected with *Chaetomium*. Among these environmental factors, pH had the greatest effect on the rhizosphere soil fungal community structure (*P* = 0.001), followed by SO_4_^2–^ (*P* = 0.014). K^+^ (*P* = 0.078), HCO_3_^–^ (*P* = 0.085), OM (*P* = 0.150), Ca_2_^+^ (*P* = 0.163), and AP (*P* = 0.174) all had a very small effect (Table 5).

## Discussion

### Rhizosphere microbial diversity of *C. salsa* parasitizing different host plant are difference

Increasingly, evidence suggests that the plant rhizosphere may recruit beneficial microbes to serve them (Dastogeer et al., 2022), but such an operating mechanism whether have an impact on the rhizosphere microenvironment of a fully parasitic plant like *Cistanche* are not fully understood. However, our results indicate there were differences in bacteria and fungi diversity of *C. salsa* parasitizing different plants. We found that fungal diversity was negatively correlated with bacterial diversity in sequencing samples. YRCR6 had the highest bacterial diversity and lowest fungi diversity, YRCR5 had the highest fungal diversity and lowest bacterial diversity. Jiao et al. (2021) and Wang et al. (2021) came up to the same result. Jiao et al. (2021) demonstrated that the balance between positive and negative bacterial-fungal associations was connected to the link between soil biodiversity and ecosystem function in complex terrestrial ecosystems. Factors such as the geographic environment and plant genotype can contribute to microbial diversity (Coleman-Derr et al., 2016), especially in natural environments where the influences are complex and diverse. However, our samples were collected from the same saline field, the same growth period, and the same soil texture. As far as possible, all samples were subjected to approximately the same factors of influence in the natural environment. Wang et al. (2021) indicated that the effect of geographical location had less influence on microbial diversity than plant cultivars. Therefore, we speculate that the differences in diversity among samples may be related to the plant cultivars. However, these differences may also be correlated with the uneven distribution of soil texture in the same area. Therefore, whether the host plant cultivars directly influences the rhizosphere bacteria and fungi diversity of *C. salsa* needs further verification. Nevertheless, our experimental results undoubtedly provide explanatory ideas for the differences in rhizosphere microbial diversity among *C. salsa* parasitizing different host plants.

### The composition of the rhizosphere microbial community was similar between samples but differed in taxonomic abundance

In the microbial community structure analysis we found no significant differences in the composition of the rhizosphere soil bacterial and fungal communities of *C. salsa* from different host plants, but there were significant differences in taxonomic abundance. Studies have shown that the composition of rhizosphere microbial communities may be the same for different plant species, but the percentage of certain microbial taxa in the community may change (Wang et al., 2019), which is consistent with our findings. As mentioned above, both soil properties and plant varieties have an impact on the composition of microbial communities. Our results showed that *Actinobacteriota*, *Chloroflexi*, *Gemmatimonadota*, and *Bacteroidota* were the dominant bacterial phyla in all samples with a total percentage greater than 69%. *Ascomycota* and *Basidiomycota* were the dominant fungal phyla in all samples with a total percentage greater than 93%. Sun et al. (2020b) concluded that the soil microbial communities for *Cistanche* are mostly tolerant to drought, salt, and alkali, which leads to the concentration of resistant microorganisms such as *Actinobacteriota* in the roots of the parasitic system of *Cistanches* to help them grow and develop. XiuLi (2021) sequenced rhizosphere soil of *Cistanche* in Ningxia, also found that *Actinobacteriota*, *Chloroflexi*, and *Bacteroidota* were the dominant bacterial phylum in the rhizosphere soil of *Cistanche*. Therefore, we speculate that this may be related to the drought and saline environment. In addition, our results showed these dominant phyla differed significantly among samples. *Actinobacteriota* was most represented in YRCR5, *Chloroflexi* was most represented in YRCR6, *Gemmatimonadota* was most represented in YRCR3, and *Bacteroidota* was most represented in YRCR1. Li et al. (2021) compared the fungi community composition of four halophyte in Xinjiang and found similarities in soil fungal community composition, but the abundance of dominant general differed significantly with plant cultivars specificity. We suspect that host plant cultivars may influence the aggregation of dominant microorganisms to produce differences in abundance. This provides clues for subsequent analysis of the rhizosphere microbial composition of *Cistanche*.

### Rhizosphere microorganisms associated with the germination of *C. salsa* are distributed in different samples

Soil microorganisms play an important role in desert ecosystems and play an important role in the growth of desert plants. Previous studies have reported that soil microbes affect parasitic plants germination, and we also found these microbes in our sequencing results. *Fusarium* spp. has been repeatedly reported to affect the germination of *Orobanchaceae* (Chen et al., 2018), and *Mortierella* are antagonistic to plant pathogens and have a great potential in the control of *Fusarium* diseases (Somers et al., 2004). We found *Mortierella* present in all of our samples and with the highest abundance in YRCR5. In addition, *Aspergillus alliaceus* affects *Orobanchaceae* seed germination (Jie et al., 2018). Our sequencing results revealed that *Aspergillus alliaceus* was present in YRCR1, YRCR2, and YRCR5 (Supplementary Figure 7), and it also has the highest abundance in YRCR5. Furthermore, *Cladosporium* can produce cotylenins to reduce the germination of *Orobanchaceae* by degrading seed germination inducers (Boari and Vurro, 2004). *Cladosporium* was found in our sequencing results with the abundance of 3.10% in YRCR5. Besides, *Cladosporium* and *Alternaria* are also common pathogens causing plant root rot, and *Alternaria* was found in YRCR1 and YRCR2 with the abundance of 8.58 and 5.67% respectively. In summary, we found that the microorganisms associated with *C. salsa* germination were more distributed in YRCR1, YRCR2, and YRCR5. We speculate that *Halocnemum strobilaceum, Atriplex patens*, and *Anabasis aphylla* as host plants may have a potentially negative impact on the germination of *C. salsa*.

### Microbial community and soil properties combine to influence plant growth and there is a potential correlation among them

Redundancy analysis of soil properties and soil microorganisms showed that the abundance of dominant bacterial and fungal communities was correlated with soil nutrients. AP was positively correlated with *norank_f__Nitrososphaeracea*, which had the highest percentage in YRCR5. AP was positively correlated with *norank_f__Nitrososphaeracea*, which had the highest percentage in YRCR5. *Nitrososphaeraceae* are important functional microorganisms involved in the Feammox process; they participate in Fe^3+^ reduction while oxidizing NH_4_^+^ to produce nitrogen gas (Li et al., 2019). This reduces the amount of soil ammonia, resulting in fewer nutrients available to the plant and thus not providing sufficient nutrients for *C. salsa* to grow. Reduction of phosphorus content can promote the secretion of strigolactone which helps parasitic plants to parasitize (Yoneyama et al., 2007). XiuLi (2021) discovered strigolactone can induce seed germination in *Orobanchaceae* and promote AMF symbiosis, thus increasing the parasitism rate of parasitic plants. We speculate that phosphorus may be involved in a negative correlation during the growth of *C. salsa*. K^+^ and PH were positively correlated with core fungal communities in YRCR6 and YRCR3 samples, and negatively correlated with fungal communities in all other samples. Potassium content can induce flue-cured tobacco to secrete large amounts of the stimulant that induces *Orobanchaceae* germination (He, 2021). Thus, it is worth thinking whether there might be some fungi in YRCR6 and YRCR3 that help *Cistanche* budding to utilize potassium and make it grow better. Generally, elevated soil PH limits the growth of fungi (Zhang et al., 2016), but in our study PH was positively correlated with *Wallemia* and *Penicillium*. This may be due to the fact that *Wallemia* is a salt-tolerant fungus (Jancic et al., 2016), and *Penicillium* readily forms mycelia and spores under alkaline conditions. Additionally, *Wallemia* metabolites exhibit resistance and cytotoxic activity and can inhibit the growth of plant pathogens on PDA plates (Chamberlin et al., 1974; Peng et al., 2011). *Penicillium* can produce antibiotic-like substances and protect plants from attack (Kawai et al., 2006). As above analysis, we can see that microorganism and soil factors each have an effect on plant growth, but they are closely related. It is worthwhile to consider in depth whether the plant will affect them. Based on the above discussion, there are significant differences in microbial community composition and soil physicochemistry in all six samples, which may be potentially related to the host plant cultivars. We speculate that there may be a potential correlation between soil physicochemical, soil microorganisms and *C. salsa* parasitic system.

## Conclusion

This study is the first time to elucidate the rhizosphere bacterial and fungal diversity and composition of *C. salsa* parasitized on different host plant by high-throughput sequencing methods. We discovered that the diversity of rhizosphere microbial communities of *C. salsa* parasitizing different plants in the same habitat was different, showing that host plant cultivars may influence the distribution of rhizosphere microbial taxa of *C. salsa*. Comparing *C. salsa* parasitized on six different host plants, we found that YRCR6 had the highest bacterial diversity and YRCR5 had the highest fungal diversity. Some microorganisms affecting the germination of *C. salsa* were found in both YRCR1, YRCR2, and YRCR5, such as, *Aspergillus alliaceus*, *Cladosporium*, and *Alternaria*. We speculate that *Halocnemum strobilaceum, Atriplex patens*, and *Anabasis aphylla* as host plants may have a potentially negative impact on the germination of *C. salsa*. This result provides an idea for the selection of host plants for *C. salsa* cultivation.

## Data availability statement

The data of high throughput sequencing in this project has been deposited in the Sequence Read Archive (SRA) of the National Center for Biotechnology Information (NCBI) under the accession number PRJNA764328.

## Author contributions

AL designed and performed the experiments, analyzed the data, and drafted the manuscript. YuL helped analyzing the data, revised, and refined the manuscript. QW and XZ performed the sample collection and analyzed part of the data. YaL participated in analyzing the dates and drafting the manuscript. JX performed the soil chemical property analysis, DNA extraction, and PCR amplification. YS and YoL designed and performed the experiments and analyzed the data. All authors read and approved the final manuscript.

## Footnotes

1
http://huttenhower.sph.harvard.edu/galaxy/root?tool_id=lefse_upload

## Supplementary material

Supplementary Figure 1 *Cistanche salsa* of different host plants. **(A)** Is *Ceratoides*, **(B)** is *Anabasis*, and **(C)** is *Atriplex.*

Supplementary Figure 2 Rarefaction curves of OTUs were clustered for a dissimilarity threshold of 3%. **(A)** Represents the rarefaction curves of the bacteria. **(B)** Represents the rarefaction curves of the fungal.

Supplementary Figure 3 Species difference analysis of all samples on genus level. The *y*-axis represents the phylum levels of species, and the *x*-axis represents the percentage of species average relative abundance in each sample group. **(A)** Is represent bacteria; **(B)** is represent Fungi. The Kruskal–Wallis rank-sum test was used to show significant differences (*: 0.01 < *P* < = 0.05, ^**^: 0.001 < *P* < = 0.01).

Supplementary Figure 4 **(A)** Is the bacterial community analysis pieplot on genus level of all samples. **(B)** Is the Fungi community analysis pieplot on genus level of all samples.

Supplementary Figure 5 Taxonomic differences were detected in different samples rhizosphere between bacteria **(A)** and fungi **(B)**. The specific classification unit name and LDA (LDA > 4.0) score were displayed in the panel.

Supplementary Figure 6 Adonis analysis account for the sample differences by different grouping factors. The “Between” boxes refer to differences between groups, while the others represent differences within their respective groups. **(A)** represenrs bacteria. **(B)** Represents fungal.

Supplementary Figure 7 The relative abundance of fungi on species level.
