# Supplementary figures and images for: Analysis of microbial diversity and community structure of rhizosphere soil of Cistanche salsa from different host plants
Source: Front Microbiol. 2022 Aug 15;13:971228. doi: 10.3389/fmicb.2022.971228 (PMC9421434; doi:10.3389/fmicb.2022.971228)

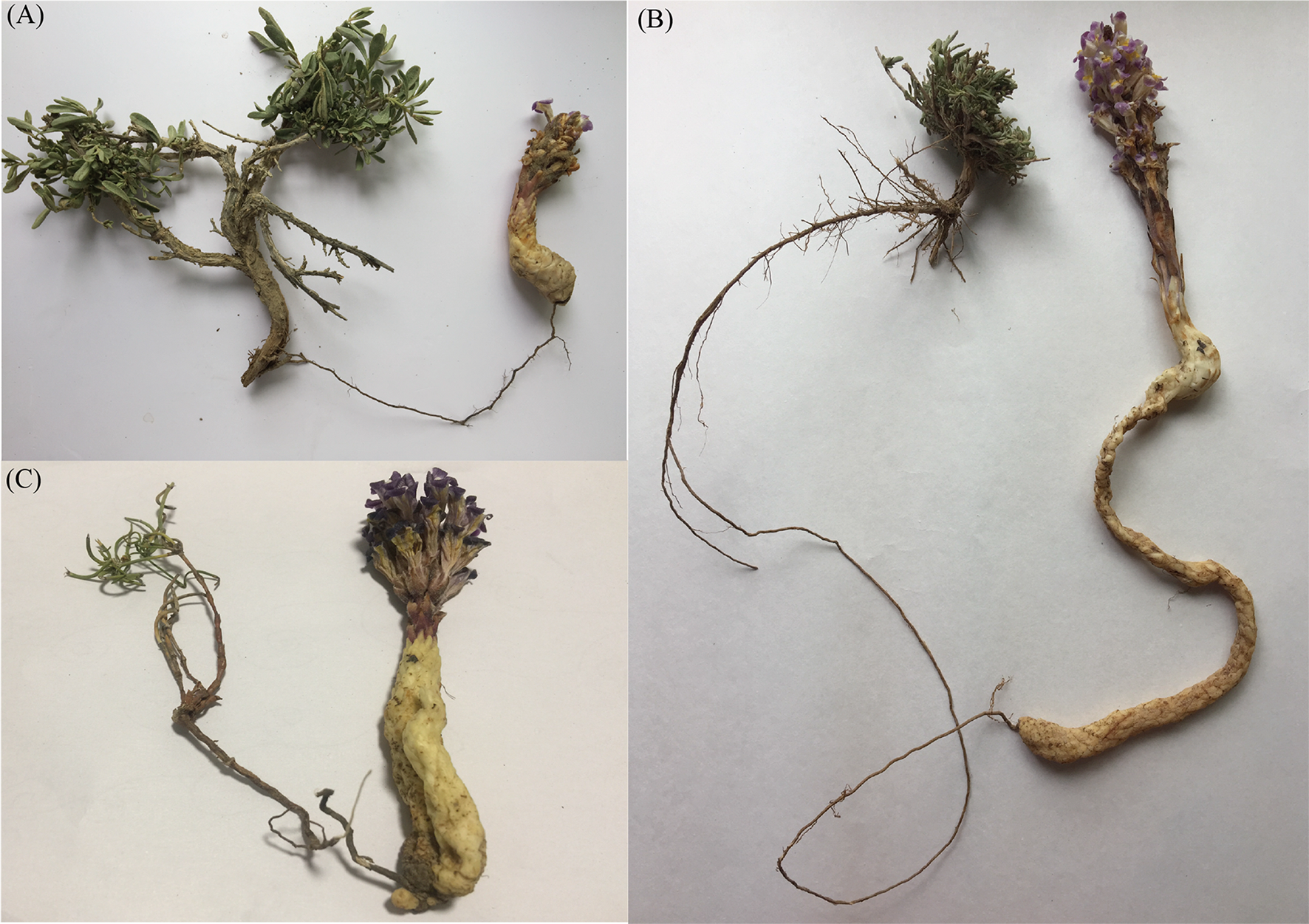

Supplement: Supplementary Figure 1 — Cistanche salsa of different host plants. (A) Is Ceratoides, (B) is Anabasis, and (C) is Atriplex. [file Image_1.TIF]

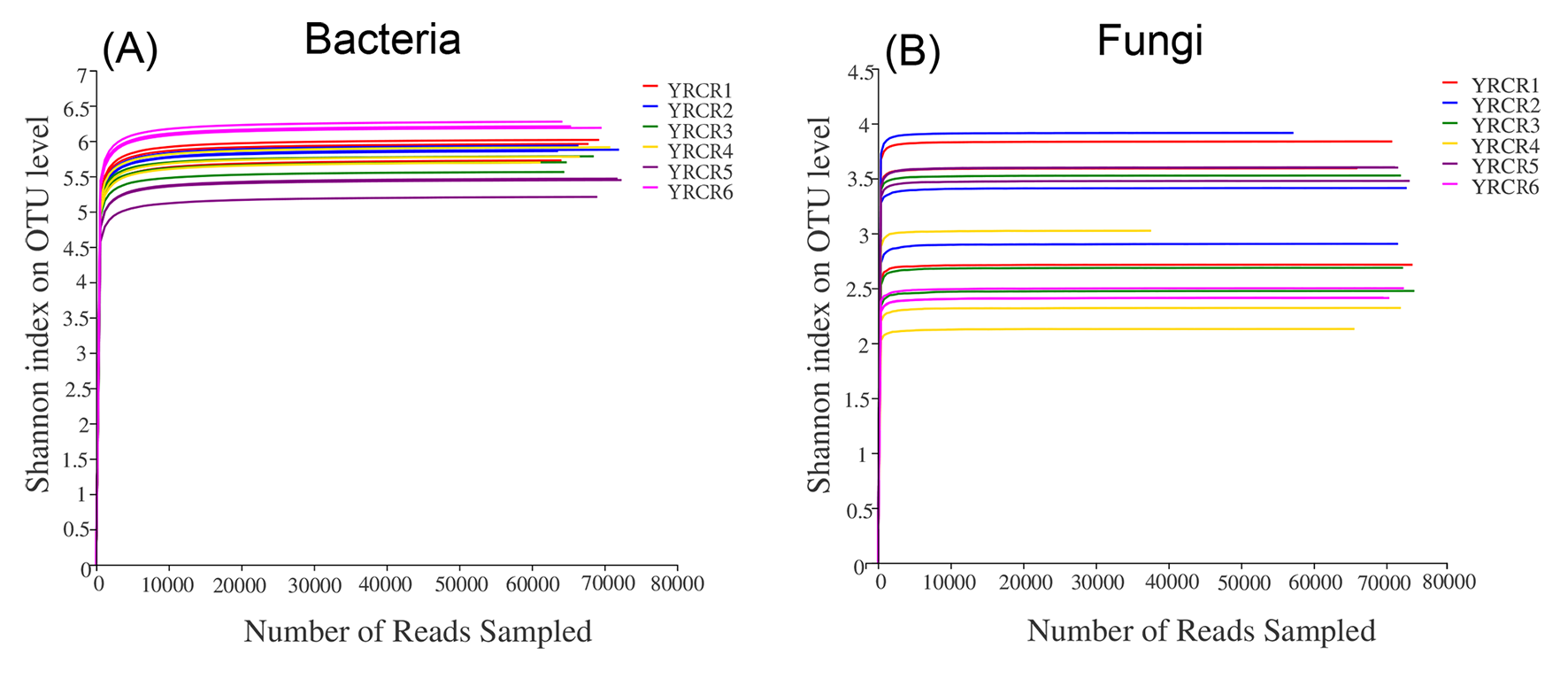

Supplement: Supplementary Figure 2 — Rarefaction curves of OTUs were clustered for a dissimilarity threshold of 3%. (A) Represents the rarefaction curves of the bacteria. (B) Represents the rarefaction curves of the fungal. [file Image_2.TIF]

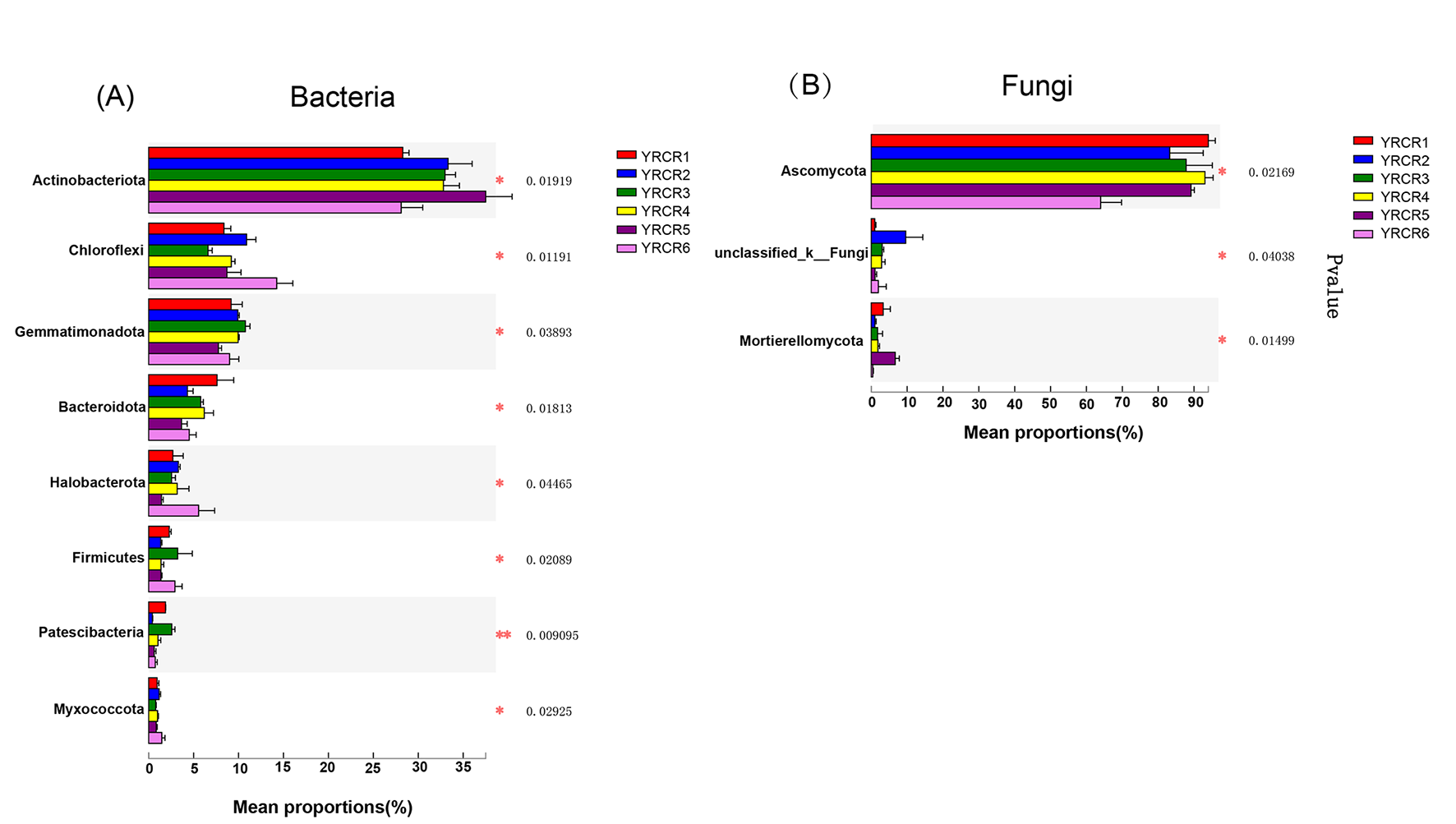

Supplement: Supplementary Figure 3 — Species difference analysis of all samples on genus level. The y-axis represents the phylum levels of species, and the x-axis represents the percentage of species average relative abundance in each sample group. (A) Is represent bacteria; (B) is represent Fungi. The Kruskal–Wallis rank-sum test was used to show significant differences (*: 0.01 < P < = 0.05, **: 0.001 < P < = 0.01). [file Image_3.TIF]

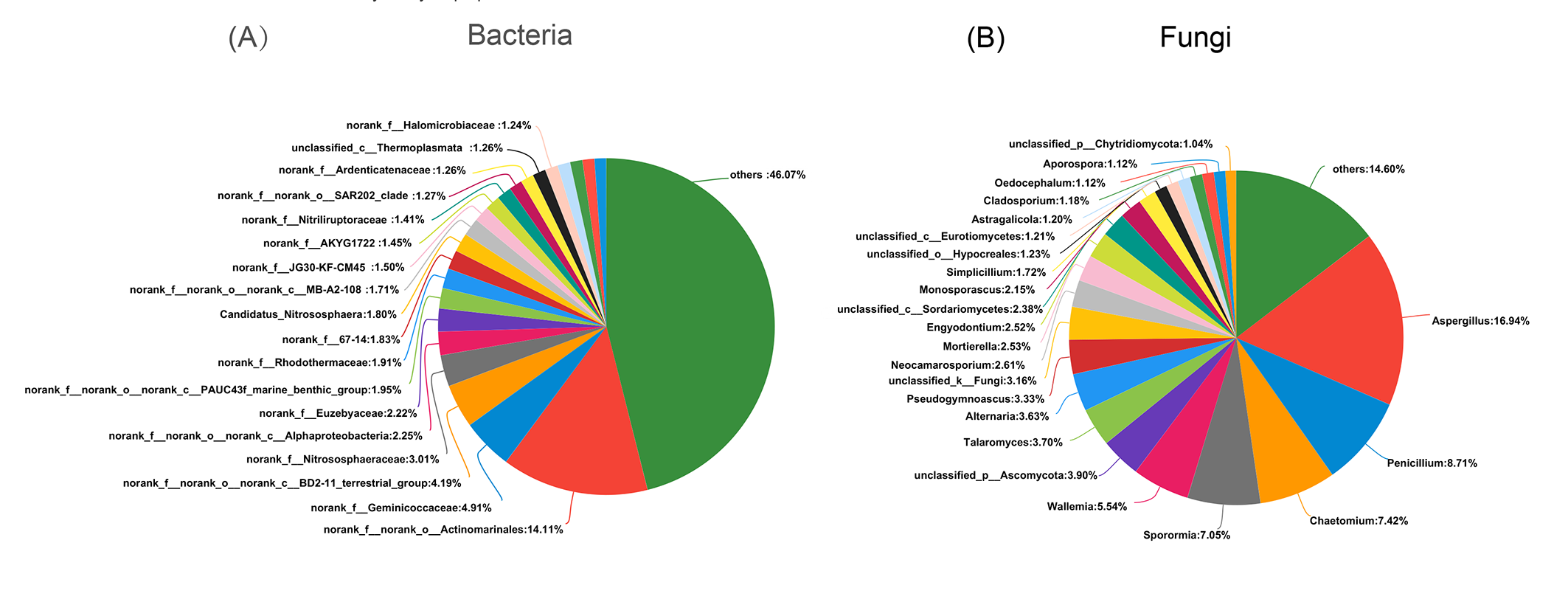

Supplement: Supplementary Figure 4 — (A) Is the bacterial community analysis pieplot on genus level of all samples. (B) Is the Fungi community analysis pieplot on genus level of all samples. [file Image_4.TIF]

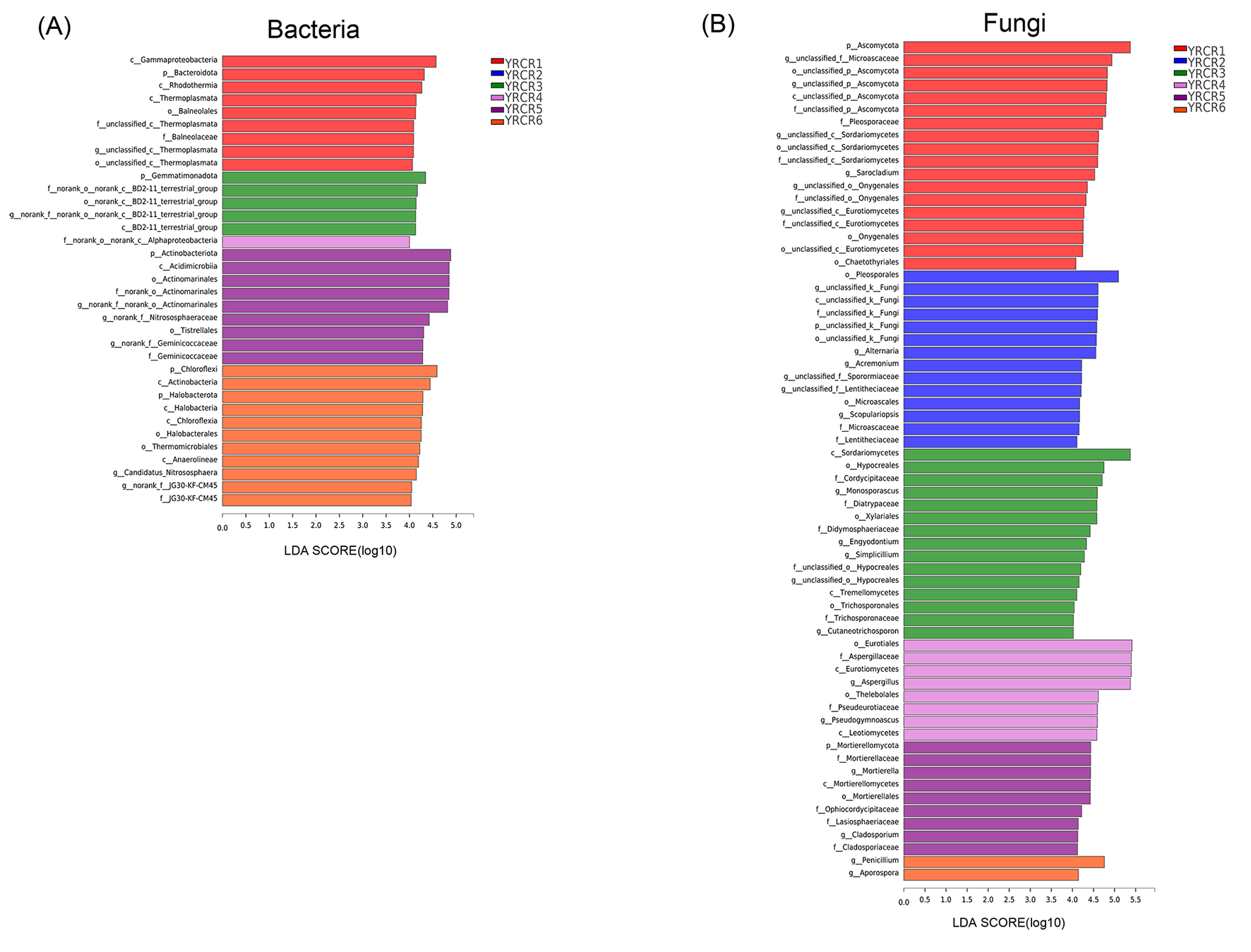

Supplement: Supplementary Figure 5 — Taxonomic differences were detected in different samples rhizosphere between bacteria (A) and fungi (B). The specific classification unit name and LDA (LDA > 4.0) score were displayed in the panel. [file Image_5.TIF]

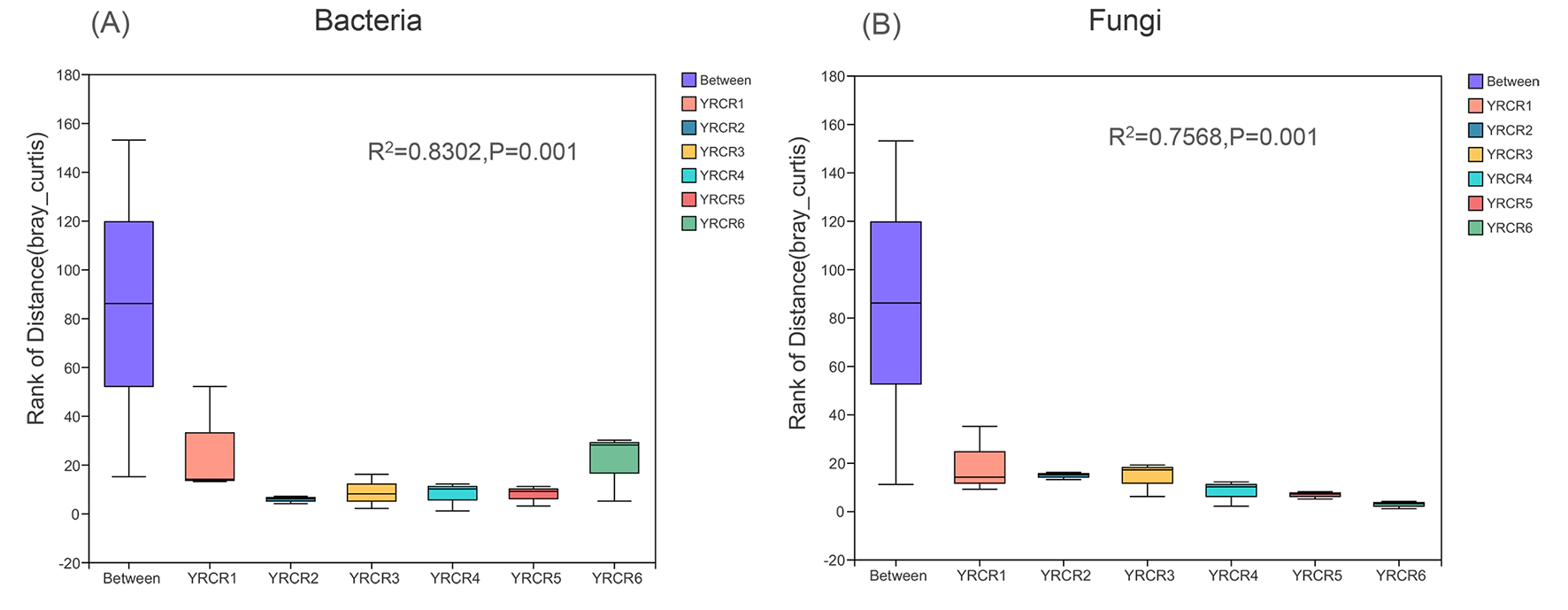

Supplement: Supplementary Figure 6 — Adonis analysis account for the sample differences by different grouping factors. The “Between” boxes refer to differences between groups, while the others represent differences within their respective groups. (A) represenrs bacteria. (B) Represents fungal. [file Image_6.TIF]

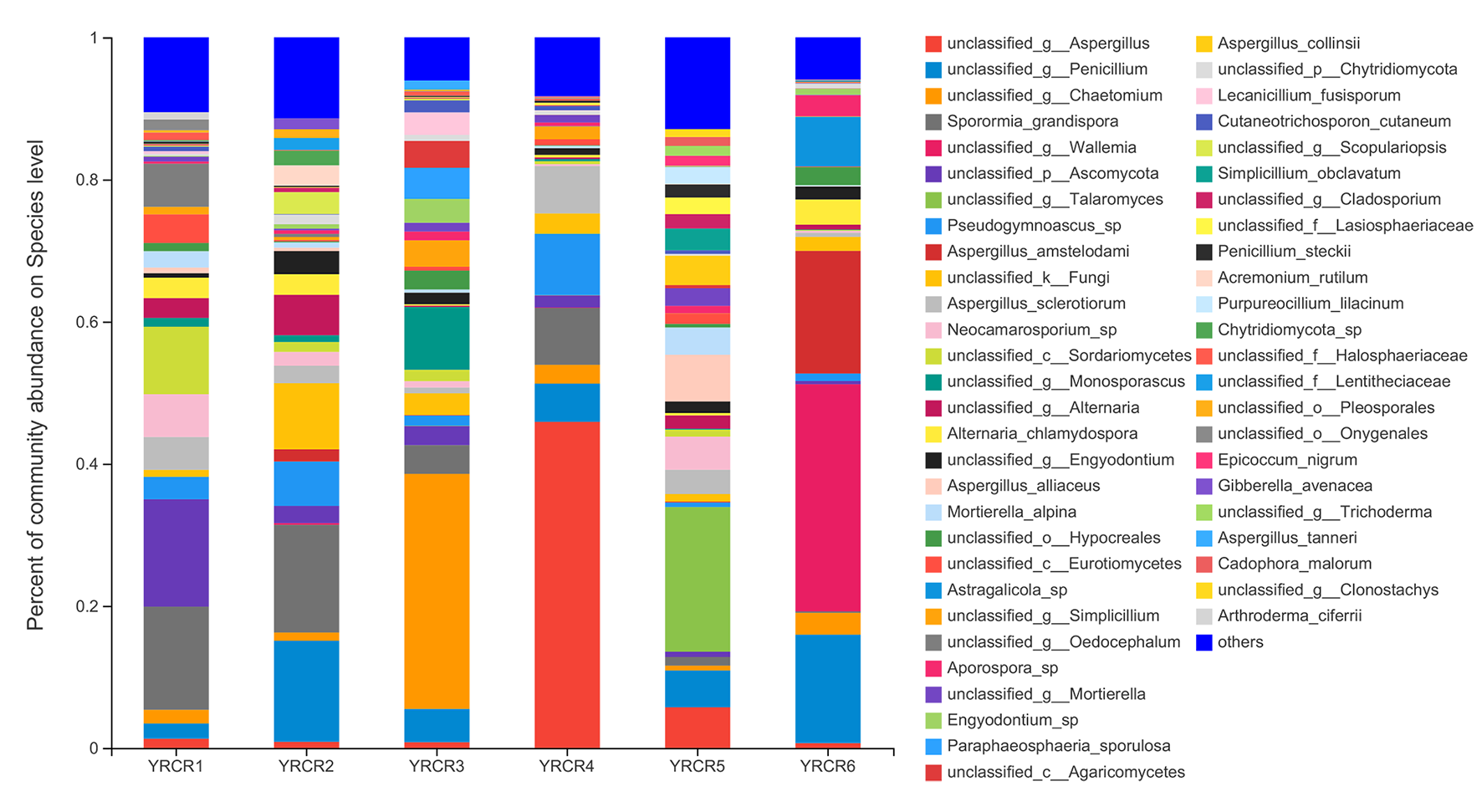

Supplement: Supplementary Figure 7 — The relative abundance of fungi on species level. [file Image_7.TIF]
